# Impaired Cardiovascular Hemodynamics in Patients Hospitalized with COVID-19 Pneumonia

**DOI:** 10.3390/jcm14061806

**Published:** 2025-03-07

**Authors:** Barbara Domino, Agnieszka Włochacz, Małgorzata Maciorowska, Krzysztof Kłos, Andrzej Chciałowski, Małgorzata Banak, Beata Uziębło-Życzkowska, Paweł Krzesiński

**Affiliations:** 1 Department of Cardiology and Internal Diseases, Military Institute of Medicine-National Research Institute, 04-141 Warsaw, Poland; ajurek@wim.mil.pl (A.W.); mmaciorowska@wim.mil.pl (M.M.); mbanak@wim.mil.pl (M.B.); buzieblo-zyczkowska@wim.mil.pl (B.U.-Ż.); pkrzesinski@wim.mil.pl (P.K.); 2 Department of Internal Diseases, Infectious Diseases and Allergology, Military Institute of Medicine-National Research Institute, 04-141 Warsaw, Poland; kklos@wim.mil.pl (K.K.); achcialowski@wim.mil.pl (A.C.)

**Keywords:** SARS-CoV-2, pneumonia, cardiovascular complications, hemodynamics, impedance cardiography, heart failure

## Abstract

**Background:** SARS-CoV-2 (severe acute respiratory syndrome coronavirus 2) infection may be associated with impaired cardiac function, especially in severe cases requiring hospitalization. Impedance cardiography (ICG) is a noninvasive method for assessing cardiac function. It could be useful for the early detection of hemodynamic dysfunction, particularly in patients with a severe course of COVID-19. **Aim:** This study aimed to analyze and compare the hemodynamic profiles of patients hospitalized with SARS-CoV-2-induced pneumonia to those of a control group. **Methods:** This prospective, observational, clinical study included 30 hospitalized patients (both men and women, mean age: 48 years) diagnosed with COVID-19 pneumonia (COVID group). Their data were compared to those of a retrospective control group (CG). The study participants were propensity score-matched based on clinical characteristics, including age, blood pressure (BP), and body mass index (BMI). ICG measurements of hemodynamic profiles were performed using a Niccomo device and included heart rate (HR), stroke volume index (SI), cardiac index (CI), velocity index (VI), acceleration index (ACI), Heather index (HI), systemic vascular resistance index (SVRI), and thoracic fluid content (TFC). **Results:** Patients with COVID-19 showed significantly higher HR (*p* < 0.0001) and SVRI (*p* = 0.0003) and lower values for several cardiac function parameters, including SI (*p* < 0.0001), VI (*p* < 0.0001), ACI (*p* = 0.004), and HI (*p* < 0.0001). Additionally, 11 patients (37%) in the COVID group had a low SI (<35 mL/m^2^), compared to only 1 patient (3%) in the control group (*p* < 0.0001). A statistically significant difference in left ventricular ejection fraction (LVEF) was also observed (*p* < 0.0001), although absolute values remained within the normal range. **Conclusions:** SARS-CoV-2 infection negatively affects the cardiovascular system, leading to impaired heart function even in low-risk patients. Impedance cardiography may serve as a simple, noninvasive tool for identifying individuals with cardiac dysfunction following COVID-19 pneumonia.

## 1. Introduction

Severe acute respiratory syndrome coronavirus 2 (SARS-CoV-2) is a highly pathogenic microorganism responsible for the COVID-19 pandemic—a global public health crisis with severe medical, humanitarian, economic, and social consequences. While coronavirus disease is primarily considered a pulmonary illness, its clinical presentation varies widely. It can range from mild upper respiratory tract inflammation to severe pneumonia, potentially leading to acute respiratory distress syndrome (ARDS) and its associated complications, including the need for respiratory support and even death.

Recent research highlights the potential impact of SARS-CoV-2 on other organs, particularly the cardiovascular system. Patients with preexisting cardiovascular conditions are considered a high-risk group with increased in-hospital mortality rates. However, early cardiovascular complications—including arrhythmias, myocarditis, acute coronary syndrome, Takotsubo syndrome, pulmonary embolism, and heart failure—have been frequently observed even in patients with no prior history of heart disease. This alarming finding has prompted further evaluation of the COVID-19 convalescent population in search for possible risk factors [1,2,3,4]. Several studies suggest that right ventricular dysfunction may result from lung involvement. Others have reported significantly elevated levels of cardiac injury biomarkers, particularly troponin, in COVID-19 patients with severe disease requiring hospitalization [1,2,3]. The pathophysiology of myocardial damage in COVID-19 is multifactorial and remains an area of ongoing research. The virus affects the lungs and other organs, including the cardiovascular system, through direct viral invasion and indirectly via a systemic inflammatory cytokine storm, with the vascular endothelium playing a key role [2,5,6]. SARS-CoV-2 can bind to angiotensin-converting enzyme 2 (ACE2), a membrane-bound amino-peptidase extensively found in the cardiovascular system, enabling it to target cells and potentially cause direct myocardial injury [2,6,7]. A high expression of ACE2 in the cardiovascular system may contribute to the increased susceptibility of patients with diagnosed cardiovascular disease to SARS-CoV-2 infection [8]. Acute cardiac injury (ACI), defined by a significant elevation in highly sensitive troponin levels, represents the most prevalent and diagnostically relevant cardiovascular complication of SARS-CoV-2 infection [8,9]. Elevated troponin levels have been strongly associated with increased mortality in COVID-19 patients [2,3,9,10,11]. Moreover, myocardial injury may serve as a substrate for the development of cardiac arrhythmias, including malignant forms such as ventricular tachycardia and ventricular fibrillation. A high frequency of arrhythmias should prompt clinical suspicion of an underlying myocardial inflammatory process [12]. This observation may, at least in part, account for the reported increase in out-of-hospital cardiac arrests observed during the COVID-19 pandemic [13]. However, it is noteworthy that analyses of in-hospital cardiac arrests in COVID-19 patients indicate a low prevalence of shockable rhythms, with asystole occurring in 89.7% of cases, pulseless electrical activity in 4.4%, and shockable rhythms in only 5.9% [14]. Furthermore, the risk of arrhythmias in COVID-19 patients may be exacerbated by concurrent electrolyte disturbances, particularly hypokalemia (low serum potassium levels) [15].

The aim of this study was to compare the hemodynamic profiles of patients recovering from COVID-19 pneumonia at hospital discharge with those of a control group to identify potential cardiovascular complications. Conventional methods for assessing hemodynamic function, such as physical examination, systolic and diastolic blood pressure measurement, and standard echocardiography, were supplemented with impedance cardiography (ICG). ICG is a noninvasive technique used to assess hemodynamic function that effectively detects early cardiovascular abnormalities [16,17].

## 2. Materials and Methods

### 2.1. Study Population

This analysis included data from 30 patients hospitalized with COVID-19 pneumonia (COVID group), who were enrolled in a prospective, observational study and compared to 30 propensity score-matched controls (CG, control group) based on clinical characteristics, including age, sex, arterial hypertension (AH) status, and body mass index (BMI).

The study was approved by the Ethical Committee of the Military Medical Institute–National Research Institute in Warsaw (No. 58/WIM/2020) and conducted in accordance with the principles of the Helsinki Declaration and Good Clinical Practice (GCP). All participants provided written informed consent before enrollment.

The COVID group consisted of adult men and women hospitalized due to COVID-19 pneumonia, confirmed by a positive polymerase chain reaction (PCR) test. Cardiovascular assessments were conducted on the day of discharge and included physical examination parameters, blood analysis (including cardiac injury biomarkers), electrocardiography, echocardiography, a six-minute walk test, and impedance cardiography.

Exclusion criteria were as follows: age >65 or <18 years, coronary heart disease, heart failure, moderate to severe heart valve disease, atrial fibrillation, a history of chronic lung or inflammatory disease, active cancer, advanced chronic kidney disease (stage 4 or 5; eGFR < 30 mL/min/1.73 m^2^), advanced hepatic dysfunction, an estimated life expectancy of less than one year, or lack of informed consent.

For the comparative analysis, 30 control participants were selected from 155 individuals enrolled in the FINE-PATH study (ClinicalTrials.gov Identifier: NCT01996085). This cohort included 120 individuals with AH who had been receiving treatment for at least 12 months, as well as 35 healthy individuals with no cardiovascular conditions or other clinically significant internal medicine disorders. The exclusion criteria for the FINE-PATH study included coronary heart disease, heart failure with reduced or mid-range ejection fraction, a history of stroke or transient ischemic attack, chronic obstructive pulmonary disease, a history of pulmonary embolism, respiratory failure (arterial partial pressure of oxygen <60 mmHg and/or partial pressure of carbon dioxide >45 mmHg), a history of head injury, pregnancy, or lack of informed consent.

### 2.2. Clinical Examination

All patients underwent a medical history interview and physical examination. The initial assessment included a detailed questionnaire regarding comorbidities, smoking history, current pharmacotherapy, and an analysis of the COVID-19 hospitalization, with a particular focus on targeted therapy and respiratory support methods. The physical evaluation encompassed anthropometric measurements (body weight, height, and BMI), heart rate (HR), office systolic and diastolic blood pressure (SBP and DBP, measured using Omron M4 Plus, Kyoto, Japan), and pulse oximetry (Oxy True FC, Selmsdorf, Germany).

### 2.3. Impedance Cardiography

A trained nurse performed impedance cardiography (ICG) measurements in a quiet environment during the morning hours, after a minimum of five minutes of rest. The examination was conducted over a 10 min resting period in a horizontal position using the Niccomo™ device (Medis, Ilmenau, Germany). The following ICG parameters were recorded and analyzed with Niccomo Software: cardiac pump function indicators—cardiac index (CI [mLm^−2^min^−1^]), stroke volume index (SI [mL/m^2^]), velocity index (VI [1000Z_0_s^−1^]), acceleration index (ACI [100Z_0_s^−2^]), and Heather index (HI [Ohm*s^2^])—and other hemodynamic parameters—thoracic fluid content (TFC [1kOhm^−1^]) and systemic vascular resistance index (SVRI [dynscm^−5^m^2^]). Blood pressure and heart rate were also measured during the ICG examination.

SI < 35 mL/m^2^ and TFC > 35 1*kOhm^−1^ were classified as abnormal, based on reports linking these thresholds to an increased risk of clinical deterioration in heart failure patients [16].

### 2.4. Echocardiography

Echocardiographic measurements were performed by an experienced cardiologist who had conducted at least 1000 examinations in the past five years. This study was carried out in a high-reference-level echocardiography laboratory in Poland using the GE Healthcare Vivid E95 ultrasound system. All parameters were assessed according to the guidelines of the European Society of Cardiology and classified as normal or abnormal based on the recommendations outlined in the publication Standardization of Adult Transthoracic Echocardiography Reporting in Agreement with Recent Chamber Quantification, Diastolic Function, and Heart Valve Disease Recommendations [18].

The echocardiographic examination protocol included the following parameters:

1. Heart chamber measurements: left ventricle end-diastolic dimension (LVDd), left ventricle end-systolic dimension (LVDs), right ventricle end-diastolic dimension (RVDd), left atrial dimension (LA), left atrial volume (LAV), and left atrial volume index (LAVi);
2. Left ventricular systolic function parameters: left ventricle ejection fraction (LVEF, assessed using the Simpson method) and left ventricle global longitudinal strain (LV GLS);
3. Left ventricular diastolic function parameters: septal annular e’ velocity (e’sept), lateral annular e’ velocity (e’lat), and left ventricular E/e’ average ratio (E/e’).

### 2.5. Statistical Methods

Statistical analysis was performed using Statistica 12.0 software (StatSoft Inc., Tulsa, OK, USA). The distribution of continuous variables was assessed visually and with the Shapiro–Wilk test. Continuous variables were presented as means ± standard deviation (SD), medians, and interquartile ranges, while categorical variables were presented in absolute values (*n*) and percentages (%). Propensity score matching was applied to select a control subgroup matched for key clinical criteria that could significantly influence the assessed values (BMI, sex, age, and the presence of AH). Differences in the absolute values of the normally distributed continuous variables were analyzed using the *t*-test, while the Mann–Whitney U-test was used for non-normally distributed variables. Categorical variables were analyzed using the chi-square test and Fisher’s exact test. A *p*-value of <0.05 was considered statistically significant.

## 3. Results

### 3.1. Baseline Characteristics

The clinical data for the COVID group are presented in Table 1. The average patient age was 48 years, and 60% were men. All patients were hospitalized due to COVID-19 pneumonia, with a median hospital stay of 12 days, and they were neither treated with antiviral drugs nor vaccinated prior to admission. During hospitalization, all patients (100%) received low-molecular-weight heparin. Dexamethasone was administered to 28 patients (93.3% of the study group), remdesivir was given to 10 patients (33.3%), and convalescent plasma was used in 16 patients (53.3%). Passive oxygen therapy (via nasal cannula or simple mask) was required in 96.3% of cases, while 6.6% required mechanical respiratory support: 3.3% received high-flow oxygen therapy, and 3.3% were placed on a ventilator. The average extent of pneumonia, as assessed by high-resolution computed tomography (HRCT), was 14%. Overweight or obesity (BMI > 25 kg/m^2^) was observed in 86.7% of COVID-19 patients, with an average BMI of 29 kg/m^2^. The mean blood pressure in the COVID group was 128/87 mmHg, with 26.7% (8 patients) having a prior diagnosis of arterial hypertension (AH), all of whom were receiving medical treatment. Cardiac biomarker levels were generally low. High-sensitivity troponin T (hs-TnT) exceeded the upper normal limit (14 ng/L) in two patients, with a maximum recorded value of 17.9 ng/L. Additionally, NT-proBNP levels exceeded the heart failure exclusion threshold (125 pg/mL) in six patients, with a maximum value of 207.6 pg/mL.

The average heart rate (HR) in the COVID group was 88 bpm. Standard echocardiography showed a normal left ventricular ejection fraction (LVEF) in all patients, with a mean value of 62%. In the control group (CG), the mean blood pressure was 119/75 mmHg, and the average HR was 67 bpm. Arterial hypertension was diagnosed in 30% of patients (nine patients). Echocardiographic assessment revealed a normal LVEF, with a mean value of 67%.

### 3.2. Intergroup Comparison

**Figure 1:**
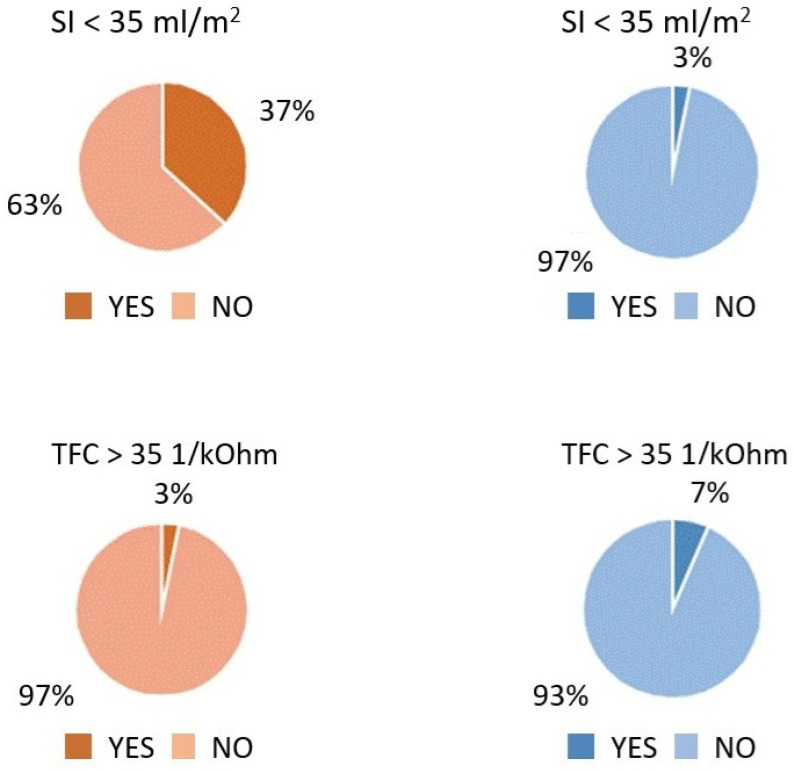
The comparison between in the COVID group (COVID, **left** graphs) and the control group (CG, **right** graphs) in the prevalence of low SI (*p* < 0.0001) and high TFC (*p* = 0.554).

The COVID-19 patients exhibited significantly higher HR (*p* < 0.0001), SBP (*p* = 0.004), DBP (*p* < 0.0001), and SVRI (*p* = 0.0003) compared to the control group. In contrast, they demonstrated lower values for key cardiac function parameters, including SI (*p* < 0.0001), VI (*p* < 0.0001), ACI (*p* = 0.004), and HI (*p* < 0.0001) (Table 2). A low SI (<35 mL/m^2^) was observed in 11 patients (37%) from the COVID-19 group, compared to only 1 patient (3%) in the control group (*p* < 0.0001). Additionally, elevated TFC (>35 1/kOhm) was detected in 1 patient (3.3%) from the COVID-19 group and 2 patients (6.6%) from the control group (Figure 1). A statistically significant difference in LVEF was also noted (*p* < 0.0001); however, absolute values remained within the normal range (Table 2). No significant intergroup differences were observed in TFC or heart chamber dimensions, including RVDd, LVDd, and LA.

## 4. Discussion

This study highlights the presence of hemodynamic dysfunction in patients shortly after hospitalization for COVID-19 pneumonia. Impedance cardiography (ICG), with its comprehensive hemodynamic assessment, revealed cardiovascular abnormalities that may be difficult to detect through standard cardiac evaluation.

In our previous study, we found no deterioration in cardiac function parameters following mild COVID-19 [19]. In the present study, we examined patients of both sexes diagnosed with SARS-CoV-2 infection, all of whom experienced significant lung involvement but had no comorbidities that could substantially impact cardiovascular function. To minimize confounding factors, we also excluded individuals with preexisting respiratory conditions.

Our findings indicate that patients recovering from COVID-19 pneumonia exhibit a higher heart rate (HR), reduced cardiac pump performance (SI, VI, ACI, HI), and increased vascular tone (SBP, DBP, SVRI). Although the left ventricular ejection fraction (LVEF) was lower in the COVID-19 patients than in the controls, it remained within the normal population range. Notably, no significant differences were observed between the groups in terms of heart chamber dimensions (RVDd, LVDd, LA) or thoracic fluid content (TFC).

To our knowledge, this is the first study investigating the use of ICG in COVID-19 patients. However, previous research has demonstrated the utility of ICG in infectious diseases, particularly sepsis. For example, Butz et al. characterized the hemodynamic profile of patients with suspected sepsis in the emergency room, showing that septic patients exhibited a lower SVRI, which increased following fluid resuscitation within one hour [20]. Similarly, Napoli et al. found that a low cardiac index (CI), as measured by ICG, was associated with an increased risk of mortality in sepsis patients [21].

Previous studies have demonstrated that impaired ICG-derived parameters of left ventricular performance (HI, ACI, VI) reflect diminished systolic and diastolic function on echocardiography. In hypertensive patients, left ventricular diastolic dysfunction was linked to a lower SI (*p* = 0.049), VI (*p* = 0.002), ACI (*p* = 0.014), and HI (*p* = 0.002) and a higher SVRI (*p* = 0.004) [22]. Another study found that patients with Cushing’s disease exhibited a significantly lower SI (*p* < 0.0001), CI (*p* < 0.0001), VI (*p* = 0.001), ACI (*p* = 0.037), and HI (*p* = 0.033) and a higher SVRI (*p* < 0.0001) than controls [23]. These parameters have also been useful in identifying subclinical cardiovascular abnormalities related to obesity [23].

In our study, we focused on convalescent patients to determine whether any lingering cardiovascular abnormalities could have long-term health implications. This hypothesis was based on known pathophysiological mechanisms. The SARS-CoV-2 spike (S) protein—specifically the S1 subunit—binds to the ACE2 receptor to enter host cells [2,7,8,9,24]. Electron microscopy and histological studies have detected viral presence not only in lung tissue but also in the kidneys, brain, liver, and heart, suggesting a tropism for highly vascularized organs [5]. Endothelial injury plays a key role in viral invasion, as SARS-CoV-2-induced endothelitis shifts endothelial function from a neutral to a pro-inflammatory and pro-thrombotic state [2,5,6]. The resulting cytokine storm is a major contributor to COVID-19-related multiorgan failure [2].

Cardiovascular complications are commonly observed in SARS-CoV-2 infection. Elevated troponin levels, indicative of myocardial injury, have been linked to more severe disease progression and increased mortality risk [2,8,9,25]. Studies estimate that myocardial injury—defined as a troponin level exceeding the 99th percentile of the upper reference limit—occurs in approximately one in four COVID-19 patients [24]. Troponin elevation typically appears around seven days after symptom onset [24] and may result from a range of causes: cardiac—acute coronary syndrome including myocardial infarction with non-obstructive coronary arteries [MINOCA], Takotsubo syndrome, arrhythmias, myocarditis, left and right heart failure, and pulmonary embolism, as well as non-cardiac conditions—e.g., sepsis or cardiovascular events [1,2,3]. Regardless of the underlying cause, elevated troponin levels are associated with poor prognosis [2,3,9]. Bois et al. reported histological evidence of myocarditis in 33% of autopsied COVID-19 patients, although the inflammatory activity was focal rather than widespread [26]. Given the limited availability of cardiac MRI and advanced echocardiographic techniques (e.g., myocardial strain imaging), the true prevalence of myocarditis in COVID-19 patients is likely underestimated.

Excessive immune activation during SARS-CoV-2 infection may also contribute to long-term cardiovascular complications, such as tissue fibrosis and vascular microangiopathy. In one study, cardiac magnetic resonance imaging performed 37–71 days after COVID-19 diagnosis revealed elevated T1 (73% of cases) and T2 (60% of cases) scores and late gadolinium enhancement (LGE) (45% of cases), suggesting persistent inflammation, fibrosis, and myocardial edema [27]. Another study found that two months post infection, nearly 80% of COVID-19 convalescents exhibited signs of myocarditis, despite most being asymptomatic and never requiring hospitalization [28]. Both direct viral cytotoxicity and immune-mediated mechanisms may contribute to the myocardial dysfunction and hemodynamic impairment detected by ICG in our study, even in the presence of a preserved LVEF.

Patients with a history of cardiovascular disease, elevated troponin or NT-proBNP levels, electrocardiographic ST segment changes, or suspected heart failure should undergo thorough cardiovascular assessment following SARS-CoV-2 infection. The European Society of Cardiology (ESC) guidelines for echocardiographic evaluation in COVID-19 patients are based on data from nearly 1300 individuals with elevated biomarkers or clinical suspicion of heart failure, revealing echocardiographic abnormalities in 50% of cases [1]. Notably, one in three echocardiograms led to a modification of treatment, while one in seven resulted in a severe diagnosis [1]. Despite these findings, there remains a critical need for noninvasive hemodynamic tools to facilitate broader screening of post-COVID patients, enabling the early identification of individuals with subclinical cardiac dysfunction who may require closer cardiological follow-up.

ICG has the potential to assist clinicians in the early identification of cardiovascular complications, even before clinical symptoms appear. This may help stratify patients who require closer cardiological follow-up, ultimately reducing the risk of developing overt cardiovascular disease. The early detection of cardiac involvement following SARS-CoV-2 infection is clinically significant, as it allows for the timely implementation of cardioprotective therapies.

### Study Limitations

The primary limitation of this study is its relatively small sample size and single-center design, primarily due to pandemic-related restrictions on diagnostic procedures and limited personnel resources. Secondly, we exclusively evaluated patients hospitalized for pneumonia, meaning our findings cannot be generalized to all individuals with SARS-CoV-2 infection, particularly those who were asymptomatic or experienced only mild symptoms. Furthermore, our study population was relatively young (patients over 65 years old were excluded) and generally healthy, with no significant comorbidities. Therefore, our results should not be considered representative of the entire population, especially the geriatric population. Additionally, the study group was too small to derive subgroups based on different treatments and to compare their effects accordingly. The last important limitation is the lack of a study prescreening list; however, only patients meeting the inclusion/exclusion criteria described in Section 2 were enrolled. The COVID-19 pandemic was a time of immense strain on the healthcare system, including our hospital, and as a result, we were forced to limit the research duties of our colleagues.

## 5. Conclusions

Although SARS-CoV-2 infection is primarily regarded as a respiratory disease, it also negatively impacts the cardiovascular system, leading to impaired heart function even in low-risk patients. Impedance cardiography may serve as a valuable, noninvasive tool for detecting early-stage cardiac dysfunction in patients recovering from COVID-19 pneumonia. The long-term persistence and prognostic significance of these abnormalities warrant further investigation.

## Figures and Tables

**Table 1:** Baseline clinical characteristics of the COVID group patients.

VARIABLE	Mean ± SD; Median (Interquartile Range) or *n* (%)
Age [years]	48.0 ± 8.8; 49 (12)
Male sex	18 (60.0)
BMI [kg/m^2^]	29.0 ± 4.1; 28.8 (6.2)
HR [bpm]	88.2 ± 14.1; 85.5 (15)
SBP [mmHg]	128.4 ± 13.3; 124.5 (20)
DBP [mmHg]	87.2 ± 8.3; 85 (12)
AH	8 (26.7)
LVEF [%]	62.9 ± 2.6; 63.5 (4)
hs-troponin T (ng/L)	7.0 ± 3.5; 6.4 (3.7)
NT-proBNP (pg/mL)	64.9 ± 62.9; 40.0 (90.7)

BMI—body mass index; HR—heart rate; SBP—systolic blood pressure; DBP—diastolic blood pressure; AH—arterial hypertension; LVEF—left ventricle ejection fraction; hs-troponin T—highly sensitive troponin T; NT-proBNP—N-terminal prohormone brain natriuretic peptide; SD—standard deviation.

**Table 2:** A comparison of routinely evaluated clinical and echocardiographic characteristics and pharmacotherapy through hemodynamic parameters between COVID group (COVID) and control group (CG).

VARIABLES	COVID Mean ± SD (Median; Interquartile Range) or *n* (%)	CG Mean ± SD (Median; Interquartile Range) or *n* (%)	*p*
Baseline clinical characteristics			
Age [years]	48.0 ± 8.8; 49 (12)	46.7 ± 8.5; 44 (12)	0.562
Male sex	18 (60.0)	20 (66.7)	0.592
BMI [kg/m^2^]	29.0 ± 4.1; 28.8 (6.2)	27.8 ± 3.6; 28.0 (5.4)	0.211
AH, n [%]	8 (26.7)	9 (30)	0.774
LVEF [%]	62.9 ± 2.6; 63.5 (4)	67.3 ± 12.2; 67 (17)	<0.0001
RVDd [mm]	29.4 ± 3.1; 30 (4)	30.0 ± 3.4; 30.5 (2.8)	0.416
LVDd [mm]	47.7 ± 4.5; 48 (6)	48.6 ± 3.8; 48.3 (5.5)	0.375
LA [mm]	38.1 ± 4.6; 38.5 (4)	36.4 ± 3.8; 35.9 (5.3)	0.087
Pharmacotherapy (discharge)			
ACEI	4 (13.3)	8 (26.7)	0.197
ARB	3 (10.0)	1 (3.3)	0.300
Beta-blocker	2 (6.7)	0 (0.0)	0.150
Diuretic	6 (20.0)	3 (10.0)	0.278
Hemodynamics (Impedance Cardiography)			
HR [bpm]	88.2 ± 14.1; 85.5 (15)	67.2 ± 12.2; 67 (17)	<0.0001
SBP [mmHg]	128.4 ± 13.3; 124.5 (20)	119.0 ± 10.8; 118.5 (12)	0.004
DBP [mmHg]	87.2 ± 8.3; 85 (12)	75.2 ± 8.2; 76 (12)	<0.0001
SI [mL·m^2^]	35.9 ± 9.3; 36.3 (13.3)	50.2 ± 8.8; 50 (17)	<0.0001
CI [mL·m^2^·min^1^]	3.12 ± 0.56; 3.1 (0.8)	3.34 ± 0.51; 3.15 (0.80)	0.122
SVRI [dyn·s·cm^5^·m^2^]	2431 ± 546; 2359 (580)	1945 ± 370; 1878 (452)	0.0003
VI [1 × 1000^1^·s^1^]	34.2 ± 10.4; 31.5 (16)	47.5 ± 10.8; 49 (23)	<0.0001
ACI [1 × 100^1^·s^2^]	52.5 ± 19.2; 46 (28)	70.8 ± 23.2; 76.5 (39)	0.002
HI [Ohm·s^2^]	8.3 ± 3.0; 7.6 (4.2)	12.9 ± 3.7; 12.8 (5.8)	<0.0001
TFC [1·kOhm^1^]	28.3 ± 3.9; 28.3 (6.6)	29.1 ± 3.8; 29.0 (5.6)	0.429

BMI—body mass index; AH—arterial hypertension; LVEF—left ventricle ejection fraction; RVDd—right ventricle end-diastolic dimension; LVDd—left ventricle end-diastolic dimension; LA—left atrial dimension; ACEI—angiotensin-converting enzyme inhibitors; ARBs—angiotensin receptor blockers; HR—heart rate; SBP—systolic blood pressure; DBP—diastolic blood pressure; SI—stroke index; CI—cardiac index; SVRI—systemic vascular resistance index; VI—velocity index; ACI—acceleration index; HI—Heather index; TFC—thoracic fluid content.

## Data Availability

The raw data supporting the conclusions of this article will be made available by the authors on request.

## References

[1] Dweck M., Bularga A., Hahn R., Bing R., Lee K., Chapman A., White A., Di Salvo G., Sade L., Pearce K. (2020). Global evaluation of echocardiography in patients with COVID-19. Eur. Heart J.—Cardiovasc. Imaging.

[2] Guzik T., Mohiddin S., Dimarco A., Patel V., Savvatis K., Marelli-Berg F., Madhur M., Tomaszewski M., Maffia P., D’Acquisto F. (2020). COVID-19 and the cardiovascular system: Implications for risk assessment, diagnosis, and treatment options. Cardiovasc. Res..

[3] Scudiero F., Silverio A., Muraca I., Russo V., DiMaio M., Silvestro A., Personeni D., Citro R., Enrico M., Galasso G. (2021). Long-term prognostic impact of right ventricular dysfunction in patients with COVID-19. J. Pers. Med..

[4] Rav-Acha M., Orlev A., Itzhaki I., Zimmerman S.F., Fteiha B., Bohm D., Kurd R., Samuel T., Asher E., Helviz Y. (2021). Cardiac arrhythmias amongst hospitalised Coronavirus 2019 (COVID-19) patients: Prevalence, characterisation, and clinical algorithm to classify arrhythmic risk. Int. J. Clin. Pract..

[5] Evans P., Rainger G., Mason J., Guzik T., Osto E., Stamataki Z., Neil D., Hoefer I., Fragiadaki M., Waltenberger M. (2020). Endothelial dysfunction in COVID-19: A position paper of the ESCWorking Group for Atherosclerosis and Vascular Biology, and the ESC Council of Basic Cardiovascular Science. Cardiovasc. Res..

[6] Pelisek J., Reutersberg B., Greber U.F., Zimmermann A. (2022). Vascular dysfunction in COVID-19 patients: Update on SARS-CoV-2 infection of endothelial cells and the role of long non-coding RNAs. Clin. Sci..

[7] Salamanna F., Maglio M., Landini M.P., Fini M. (2020). Body Localization of ACE-2: On the Trail of the Keyhole of SARS-CoV-2. Front. Med..

[8] Ielapi N., Licastro N., Provenzano M., Andreucci M., de Franciscis S., Serra R. (2020). Cardiovascular disease as a biomarker for an increased risk of COVID-19 infection and related poor prognosis. Biomark. Med..

[9] Bansal M. (2020). Cardiovascular disease and COVID-19. Diabetes Metab. Syndr..

[10] Shi S., Qin M., Shen B., Cai Y., Liu T., Yang F., Gong W., Liu X., Liang J., Zhao Q. (2020). Association of Cardiac Injury With Mortality in Hospitalized Patients with COVID-19 in Wuhan, China. JAMA Cardiol..

[11] Guo T., Fan Y., Chen M., Wu X., Zhang L., He T., Wang H., Wan J., Wang X., Lu Z. (2020). Cardiovascular Implications of Fatal Outcomes of Patients with Coronavirus Disease 2019 (COVID-19). JAMA Cardiol..

[12] Ranard L.S., Fried J.A., Abdalla M., Anstey D.E., Givens R.C., Kumaraiah D., Kodali S.K., Takeda K., Karmpaliotis D., Rabbani L.E. (2020). Approach to Acute Cardiovascular Complications in COVID-19 Infection. Circ. Heart Fail..

[13] Baldi E., Sechi G.M., Mare C., Canevari F., Brancaglione A., Primi R., Klersy C., Palo A., Contri E., Ronchi V. (2020). Out-of-Hospital Cardiac Arrest during the COVID-19 Outbreak in Italy. N. Engl. J. Med..

[14] Shao F., Xu S., Ma X., Xu Z., Lyu J., Ng M., Cui H., Yu C., Zhang Q., Sun P. (2020). In-hospital cardiac arrest outcomes among patients with COVID-19 pneumonia in Wuhan, China. Resuscitation.

[15] Chen D., Li X., Song Q., Hu C., Su F., Dai J., Ye Y., Huang J., Zhang X. (2020). Assessment of Hypokalemia and Clinical Characteristics in Patients with Coronavirus Disease 2019 in Wenzhou, China. JAMA Netw. Open.

[16] Packer M., Abraham W., Mehra M., Yancy C., Lawless C., Mitchell J., Smart F., Bijou R., O’Connor C., Massie B. (2006). Utility of impedance cardiography for the identification of short-term risk of clinical decompensation in stable patients with chronic heart failure. J. Am. Coll. Cardiol..

[17] Krzesiński P., Gielerak G., Kowal J. (2009). Impedance cardiography—A modern tool for monitoring therapy of cardiovascular diseases. Kardiol. Pol..

[18] Galderisi M., Cosyns B., Edvardsen T., Cardim N., Delgado V., Di Salvo G., Donal E., Sade L.E., Ernande L., Garbi M. (2017). Standardization of adult transthoracic echocardiography reporting in agreement with recent chamber quantification, diastolic function, and heart valve disease recommendations: An expert consensus document of the European Association of Cardiovascular Imaging. Eur. Heart J.-Cardiovasc. Imaging.

[19] Uziębło-Życzkowska B., Krzesiński P., Domino B., Chciałowski A., Maciorowska M., Gielerak G. (2022). Echocardiographic assessment of cardiac function after mild coronavirus disease 2019: A preliminary report. J. Clin. Ultrasound.

[20] Butz J., Shan Y., Samayoa A., Kirton O., Vu T. (2019). The utility of impedance cardiography in hemodynamic monitoring of patients with sepsis. Trauma Surg. Acute Care Open.

[21] Napoli A., Machan J., Corl K., Forcada A. (2010). The use of impedance cardiography in predicting mortality in emergency department patients with severe sepsis and septic shock. Acad. Emerg. Med..

[22] Krzesiński P., Gielerak G., Stańczyk A., Uziębło-Życzkowska B., Smurzyński P., Piotrowicz K., Skrobowski A. (2015). What does impedance cardiography add more to the assessment of left ventricular diastolic function in essential hypertension?. Pol. Merkur. Lekarski.

[23] Jurek A., Krzesiński P., Gielerak G., Witek P., Zieliński G., Kazimierczak A., Wierzbowski R., Banak M., Uziębło-Życzkowska B. (2021). Cushing’s Disease: Assessment of Early Cardiovascular Hemodynamic Dysfunction with Impedance Cardiography. Front. Endocrinol..

[24] Cosyns B., Lochy S., Luchian M., Gimelli A., Pontone G., Allard S., Mey J., Rosseel P., Dweck M., Petersen S. (2020). The role of cardiovascular imaging for myocardial injury in hospitalized COVID-19 patients. Eur. Heart J.—Cardiovasc. Imaging.

[25] Urban S., Fułek M., Błaziak M., Iwanek G., Jura M., Fułek K., Guzik M., Garus M., Gajewski P., Lewandowski Ł. (2022). COVID-19 Related Myocarditis in Adults: A Systematic Review of Case Reports. J. Clin. Med..

[26] Bois M., Boire N., Layman A., Aubry M., Alexander M., Roden A., Hagen C., Quinton R., Larsen C., Erben Y. (2021). COVID-19-associated Non-Occlusive Fibrin Microthrombi in the Heart. Circulation.

[27] Atri L., Morgan M., Harrell S., AlJaroudi W., Berman A.E. (2021). Role of cardiac magnetic resonance imaging in the diagnosis and management of COVID 19 related myocarditis: Clinical and imaging considetions. World J. Radiol..

[28] Puntmann V.O., Carerj M.L., Wieters I., Fahim M., Arendt C., Hoffmann J., Shchendrygina A., Escher F., Vasa-Nicotera M., Zeiher A.M. (2020). Outcomes of cardiovascular magnetic resonance imaging in patients recently recovered from coronavirus disease 2019 (COVID-19). JAMA Cardiol..

